# Fixation methods can differentially affect ciliary protein immunolabeling

**DOI:** 10.1186/s13630-017-0045-9

**Published:** 2017-03-24

**Authors:** Kiet Hua, Russell J. Ferland

**Affiliations:** 1 Department of Neuroscience and Experimental Therapeutics, Albany Medical College, 47 New Scotland Avenue, MC-136, Albany, NY 12208 USA; 2 Department of Neurology, Albany Medical College, Albany, NY 12208 USA

**Keywords:** Primary cilia, Immunocytochemistry, Fixation, Technique

## Abstract

**Background:**

Primary cilia are immotile, microtubule-based organelles present on most cells. Defects in primary cilia presence/function result in a category of developmental diseases referred to as ciliopathies. As the cilia field progresses, there is a need to consider both the ciliary and extraciliary roles of cilia proteins. However, traditional fixation methods are not always suitable for examining the full range of localizations of cilia proteins. Here, we tested a variety of fixation methods with commonly used cilia markers to determine the most appropriate fixation method for different cilia proteins.

**Methods:**

Mouse inner medullary collecting duct and human retinal pigmented epithelial cells were grown to confluence, serum starved, and fixed with one of the following fixation agents: paraformaldehyde–sucrose, paraformaldehyde–PBS, methanol, cytoskeletal buffer followed by methanol, or three variations of cytoskeletal buffer–paraformaldehyde fixation. Each cell type and fixation method combination was probed with the following ciliary markers: acetylated α-tubulin, detyrosinated tubulin, polyglutamylated tubulin, β-tubulin, adenylyl cyclase 3 (AC3), ADP-ribosylation factor-like protein 13b (Arl13b), centrosome and spindle pole associated protein 1 (CSPP1), or intraflagellar transport protein 20 (IFT20). Intraflagellar transport protein 88 (IFT88) and GM130 (Golgi marker) were also used. We assessed actin (via phalloidin) and microtubule integrity, centrioles, cilia, and two extraciliary sites (mitotic figures and Golgi).

**Results:**

For the cilia markers examined, paraformaldehyde fixation preserved cilia immunolabeling of cilia-membrane proteins (AC3 and Arl13b), but failed to reveal cilia immunostaining of axonemal proteins (CSPP1 and IFT20). Methanol revealed cilia labeling for some axonemal proteins, but not others, and this depended on cell type. Generally, any method that first included a wash in cytoskeletal buffer, before fixing, revealed more distinct cilia immunolabeling for axonemal proteins (CSPP1, IFT20, and IFT88), but resulted in the loss of cilia labeling for cilia-membrane proteins (AC3 and Arl13b). All three different post-translational modifications of tubulin antibodies positively immunolabeled cilia in all fixation methods tested. Ultimately, we found that fixing cells in a solution of paraformaldehyde prepared in cytoskeletal buffer allowed for the preservation of cilia immunolabeling for most cilia proteins tested and allowed visualization of two extraciliary sites (mitotic figures and Golgi).

**Conclusion:**

Some general patterns were observed to guide in the choice of a fixation agent. Cilia-membrane proteins generally benefit from quick fixation with no prior permeabilization, whereas axonemal proteins tend to benefit from permeabilization and use of cytoskeletal buffer.

## Background

Primary cilia are immotile, usually found singularly per cell, and are recognized for their roles in signaling and development [1]. Structurally, the primary cilium is composed of the basal body and the axoneme [1–4]. The basal body is a mature mother centriole that has docked to the plasma membrane, and it gives rise to the microtubules that form the cilium [5]. These microtubules form the shaft or axoneme of the cilium, and are arranged in a 9+0 pattern that consists of 9 doublets of microtubules arranged in a circular fashion [6]. This is in contrast to motile cilia which have a 9+2 pattern that consists of 9 microtubule doublets surrounding a central pair of microtubules [6]. Primary cilia are organelles and represent a separate compartment of the cell, meaning that the cilia-membrane and cilioplasm are distinct from the plasma membrane and cytoplasm, respectively [7]. The cilium also has its own transport system, the intraflagellar transport system (IFT), consisting of motor protein complexes that carry proteins in an anterograde and retrograde manner along the microtubular axoneme [8]. A defect in any of the proteins important for the assembly, maintenance and/or function of the primary cilium can result in a category of developmental diseases called ciliopathies [1, 2, 9].

The history leading to our current understanding of the primary cilium is partly dependent on technological advances. Primary cilia were first observed in 1898 by the Swiss anatomist, KW Zimmerman, who drew images that depicted the mother and daughter centrioles with a primary cilium protruding into the luminal space of a kidney tubule [10, 11]. Zimmerman noticed that this immotile structure was found one per cell, and named it the “centralgeissel”, meaning the central flagellum, and surmised that it had a sensory function [11]. However, it was not until the invention of the electron microscope that this organelle was verified [10, 12]. In 1985, Poole et al. speculated that primary cilia have chemical and sensory roles [13], assumptions that have now been verified by multiple labs [14–17]. Here, we suggest that an important subcategorization within the primary cilia field will lie in the study of primary cilia proteins at extraciliary sites [18].

Many cilia proteins localize to cellular sites besides the cilium [18]. For example, centrosome and spindle pole associated protein 1 (CSPP1) was initially described as a centrosome and mitotic spindle protein [19], and was later found to also localize in primary cilia [20]. CSPP1 has now also been found to localize to desmosomes [21] and kinetochores [22]. Arl13b is not only found in cilia, but also co-labels with endocytic markers [23]. Intraflagellar transport protein 20 (IFT20) is another cilia marker, and it also localizes to the Golgi [24]. Therefore, further research is needed to understand the role of cilia proteins at extraciliary sites and how this might contribute to the underlying pathologies of ciliopathies [18].

Both the microtubule and actin cytoskeleton have been shown to be critical for proper primary cilia formation/function. It is not surprising that the microtubule cytoskeleton plays a role in ciliogenesis as the cilium is a microtubule-based structure. In fact, CSPP1, a ciliogenesis protein, has a microtubule-binding domain [25]. Defects in another ciliogenesis protein, Ahi1, have also been reported to result in a disorganized microtubule cytoskeleton [26], suggesting that Ahi1 may have a role in microtubule organization. Moreover, knockdown of Ahi1 in IMCD3 cells was shown to result in a disorganized actin network as well [26]. Actin proteins are now increasingly being shown to be important for ciliogenesis. Recently, multiple labs reported that non-muscle myosin heavy chain 10 (MYH10), an actin regulating protein, is also necessary for ciliogenesis [27, 28]. Interestingly, MYH10 does not localize to the basal body or axoneme of primary cilia, but loss of MYH10 results in loss of cilia [27]. This suggests (1) that proper functioning of the microtubule and actin cytoskeleton is necessary for the construction of the primary cilium, and/or (2) that at least some cilia proteins also function as more general cytoskeletal proteins (i.e., regulators of actin and microtubules). Consequently, understanding how different fixation techniques alter the actin and microtubule cytoskeleton, as well as the primary cilium, is critical for understanding ciliopathies.

The effect of fixation on ciliary protein localization via immunocytochemistry can be demonstrated with the cilia-associated protein, CSPP1 [20]. CSPP1 was initially identified as a protein that localizes to centrosomes and mitotic spindles [19]. Subsequently, CSPP1 was found to localize to primary cilia in methanol-fixed cells [20]. The fixation process used is important because paraformaldehyde fixation does not reliably yield cilia immunolabeling when using the same CSPP1 antibody (unpublished observations). Paraformaldehyde is known to disrupt the native conformation of microtubules and can hide cilia immunostaining for some cilia markers [29]. This problem can sometimes be mitigated by use of methanol as a fixation agent; however, methanol obscures the phalloidin epitope (a widely used reagent to view actin stress fibers). Therefore, alternative fixation methods are necessary to allow for the reliable, concurrent viewing of microtubules, phalloidin-stained actin stress fibers, and cilia markers.

Preservation of the cytoskeleton during fixation has historically been achieved through the use of cytoskeleton buffers [30]. In vitro observations show that tubulin polymerized when (1) calcium is absent, (2) magnesium is added to the buffer, and (3) when the incubation occurred at 35 °C as opposed to 0 °C [31]. This led to the development and use of various forms of cytoskeletal buffers (also referred to as extraction buffers) that consisted of EGTA (a calcium chelator), magnesium, and Triton X-100 detergent for extraction [32]. These cytoskeleton buffers proved useful for studying CSPP1 when in 2014, three laboratories published papers that showed *CSPP1* was a causative gene for Joubert syndrome (a neurodevelopmental ciliopathy). However, there were discrepancies with two of the laboratories reporting different immunolabeling patterns for CSPP1 [33, 34]. Both laboratories used primary human dermal fibroblasts collected from control subjects and individuals with Joubert syndrome. One laboratory showed CSPP1 localization to the centrosomes [33], while our laboratory observed CSPP1 localization also at the axoneme of the primary cilium [34]. We found that when studying CSPP1, a microtubule-stabilizing buffer similar to cytoskeletal buffer was required to reveal consistent and reliable CSPP1 labeling at the ciliary axoneme [34]. These studies indicate that careful consideration and understanding of fixation methods are important for interpreting localizations of ciliary proteins. For that reason, we explored the advantages and disadvantages of various fixation methods in a systematic and comprehensive attempt to elucidate how these different methods affect immunolabeling of popularly used cilia markers. Our results would advocate the use of cytoskeletal buffers during cell fixation, which largely preserves labeling of cilia, microtubules, actin stress fibers, and at least the two extraciliary sites we examined, mitotic figures and Golgi.

## Methods

### Cell culture

Mouse inner medullary collecting duct (IMCD3) cells and human retinal pigmented epithelial (RPE) cells were grown in Dulbecco’s modified Eagle’s medium/nutrient mixture F12 (DMEM/F12; Sigma, D8437) and Dulbecco’s modified Eagle’s medium (DMEM; Sigma, D5796), respectively. In both cases, media were supplemented with 10% fetal bovine serum (FBS; Hyclone, SH30070.03) and 1% penicillin–streptomycin (Gibco, 15140-122). For immunolabeling studies, cells were trypsinized (0.25%), seeded, and grown on 12-mm glass coverslips until confluent in a 37 °C incubator with 5% CO_2_. Upon reaching confluence, cells were switched to starvation medium (DMEM/F12 or DMEM supplemented with 1% penicillin–streptomycin, but 0% FBS) for 24 h to induce robust ciliogenesis.

### Fixation methods

#### Paraformaldehyde (PFA)

Paraformaldehyde was made using powdered PFA (Sigma, P6148) that was always stored at 4 °C, and dissolved into phosphate-buffered saline (PBS) to a final concentration of 4%. The pH of the PFA solution was 7.0. At the beginning of the experiment, a large batch of PFA–sucrose and PFA–PBS was prepared, aliquoted, and frozen at −20 °C so that all experiments could be performed with PFA prepared from the same batch. PFA was always stored at −20 °C, and only thawed out in aliquots when needed. Aliquots were never used for longer than 1 day after being thawed.

#### Paraformaldehyde–PBS (PFA–PBS)

Cells were first washed in PBS, and then fixed for 10 min at room temperature in a solution of 4% PFA prepared in PBS. Cells were again extensively washed, and subsequently blocked for 1 h in 1% bovine serum albumin (BSA; Sigma, A7030) prepared in Banker’s PBS [140 mM NaCl (Sigma, S9888), 15 mM phosphate buffer] [35] with 0.1% Triton X-100 (PBS-Tx; Sigma, T9284) for 1 h at room temperature.

#### Paraformaldehyde–sucrose (PFA-S)

Cells were washed in PBS, and then fixed at room temperature for 10 min in a solution of 4% PFA prepared in PBS and 4% sucrose (Sigma, S0389). Cells were extensively washed again, and then blocked in 1% BSA/Banker’s PBS-Tx (0.1%) for 1 h at room temperature.

#### Methanol (MeOH)

Cells were first washed in PBS, and then fixed with cold MeOH (−20 °C; Absolute–Acetone Free; Sigma, M1775) inside a −20 °C freezer for 10 min. The plate of cells to be fixed was placed directly onto the freezer coils for maximum coldness. After being washed again, cells were blocked for 1 h at room temperature in 1% BSA/Banker’s PBS-Tx (0.1%).

#### Cytoskeletal buffer (CB) for fixation

Cytoskeletal buffer was prepared with the following components: 100 mM NaCl (Sigma, S9888), 300 mM sucrose (Sigma, S0389), 3 mM MgCl_2_ (Sigma, M2670), and 10 mM PIPES (Sigma, P6757). The pH of this solution was adjusted to 6.9. The final CB solution was filtered and stored at −20 °C until needed. CB was not used for more than 1 day after thawing. Immediately before use, for a 50 ml volume of CB, 250 µl of Triton X-100 and 250 µl of EGTA (1 M) (Sigma, E3889) were added. The CB was warmed to 37 °C in a water bath before use. It should be noted that any fixation using a CB wash must be done quickly or cells will float off the coverslip.

#### Cytoskeletal buffer followed by methanol (CB → MeOH)

Cells were quickly washed with cytoskeletal buffer, then immediately subjected to fixation with cold MeOH inside a −20 °C freezer. Cells were then washed and blocked in a 1% BSA/Banker’s PBS-Tx (0.1%) solution for 1 h.

#### 4% paraformaldehyde prepared in cytoskeletal buffer (CB-PFA)

Four percent PFA was prepared in CB. After washing with PBS, cells were fixed for 10 min at 37 °C with CB-PFA. Cells were extensively washed and blocked for 1 h at room temperature in 1% BSA/Banker’s PBS-Tx (0.1%).

#### Cytoskeletal buffer wash followed by 4% paraformaldehyde prepared in cytoskeletal buffer (CB → CB-PFA)

Cells were washed quickly two times with pre-warmed CB. CB should be applied, and then removed within a few seconds (longer incubations will result in cells floating off the coverslips). Cells were then fixed with CB-PFA for 10 min at 37 °C. Cells were extensively washed and blocked for 1 h at room temperature in 1% BSA/Banker’s PBS-Tx (0.1%).

#### Cytoskeletal buffer wash, followed by 4% paraformaldehyde prepared in cytoskeletal buffer, and a final post-fixation in methanol (CB → CB-PFA → MeOH)

Cells were washed twice with pre-warmed CB, fixed for 10 min in CB-PFA, and then post-fixed in ice-cold MeOH (−20 °C) for an additional 10 min in a −20 °C freezer. Then, cells were extensively washed and blocked for 1 h at room temperature in 1% BSA/Banker’s PBS-Tx (0.1%).

#### Pre- and post-fixation permeabilization

For these experiments, cells were washed in PBS, and then fixed for 10 min in PFA-S as described above, but were additionally subjected to a permeabilization step either before or after being fixed. The following were used for permeabilization: Banker’s PBS with 0, 0.04, 0.1, 0.5, 1% Triton X-100, or CB. For pre-fixation permeabilization, cells were simply washed quickly with the permeabilization solutions before being fixed. These washes must be done quickly to avoid cells detaching from the coverslip. For post-fixation permeabilization, cells were fixed first in PFA-S, and then permeabilized with one of the permeabilization solutions. In both cases, cells were later extensively washed, blocked, and incubated in solutions containing the same concentration of Triton X-100 as their treatment group, with the exception of the CB group, which was treated with 0.1% Triton X-100 for all post-fixation steps.

### Immunofluorescence

After the above fixation methods, cells were incubated in a primary antibody solution prepared in 1% BSA and Banker’s PBS-Tx (different concentrations of Triton X-100 were used depending on the experiment) overnight at 4 °C. Primary antibodies used, included rabbit anti-β-tubulin (1:1000, Abcam, ab6046), mouse anti-acetylated α-tubulin (1:50,000, Sigma, clone 6-11B-1), rabbit anti-detyrosinated tubulin (1:500, Millipore, AB3201), mouse anti-polyglutamylated tubulin (1:500, Sigma, T9822), rabbit anti-adenylyl cyclase 3 (AC3, 1:200, Santa Cruz Biotechnology, sc-588), rabbit anti-ADP-ribosylation factor-like protein 13b (Arl13b, 1:200, UC Davis/NIH NeuroMab Facility clone N295B/66), rabbit anti-centrosome and spindle pole associated protein 1 (CSPP1, 1:200, Proteintech, 11931-1-AP), rabbit anti-intraflagellar transport protein 20 (IFT20, 1:200, Sigma, HPA021376), rabbit anti-intraflagellar transport protein 88 (IFT88, 1:200, Proteintech, 13967-1-AP), and mouse anti-Golgi matrix protein 130 (GM130, 1:1,000, BD Bioscience, 610822). After extensive washing, cells were then incubated in either mouse or rabbit Alexafluor 488 and Alexafluor 546 secondary antibodies (1:500, Life Technologies) for 1 h at room temperature. Last, nuclei were labeled with Hoechst 33258 (1 µg/ml), and actin was labeled with phalloidin-546 (1:200, Sigma, A22283) when appropriate. All coverslips were mounted with Fluoromount G (Southern Biotech, 0100-01). Images were obtained with a Zeiss AxioImager.Z1 upright microscope equipped with an AxioCam MRm camera, using a 63× plan-apochromat (1.4 NA) oil objective (Zeiss) and fluorescent filter sets 20, 34, 38HE, and 50 (Zeiss). Images were processed with AxioVision Rel. 4.5 software, and imported into ImageJ [36] and/or Adobe Photoshop CS6 version 13.0 × 64 to assemble montages.

## Results

### Different fixation methods affect microtubule immunolabeling and phalloidin staining of actin stress fibers

**Fig. 1:**
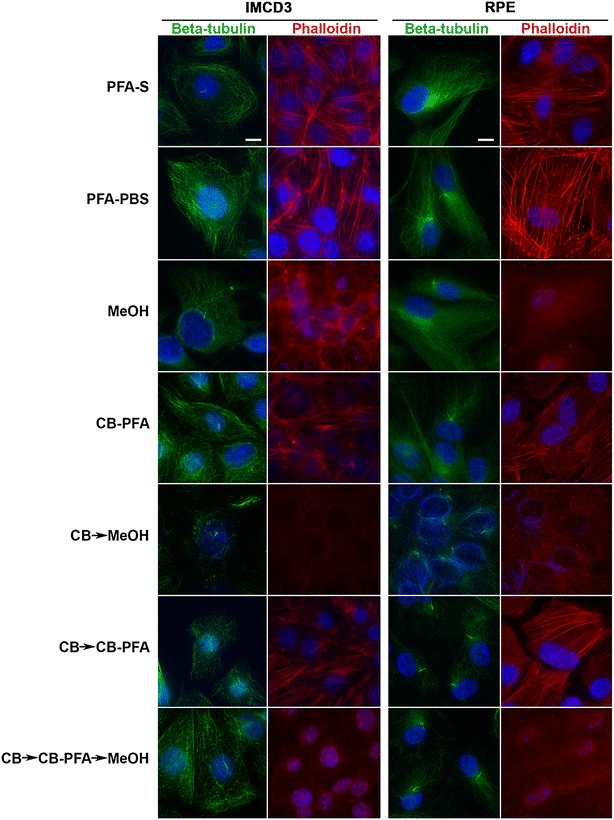
Different fixation methods affect microtubule immunolabeling and phalloidin staining of actin stress fibers in IMCD3 and RPE cells. IMCD3 and RPE cells, subjected to various fixation methods, were labeled for microtubules (β-tubulin, *green*), actin stress fibers (phalloidin, *red*), and nuclei staining (Hoechst, *blue*). Cilia and microtubules were preserved in both IMCD3 and RPE cells to varying extents in all fixation methods. Phalloidin staining of stress fibers was not preserved in the MeOH, CB → MeOH, and the CB → CBPFA → MeOH groups for both cell lines, but was preserved in all other fixation groups. *Scale bars* 10 µm

**Fig. 2:**
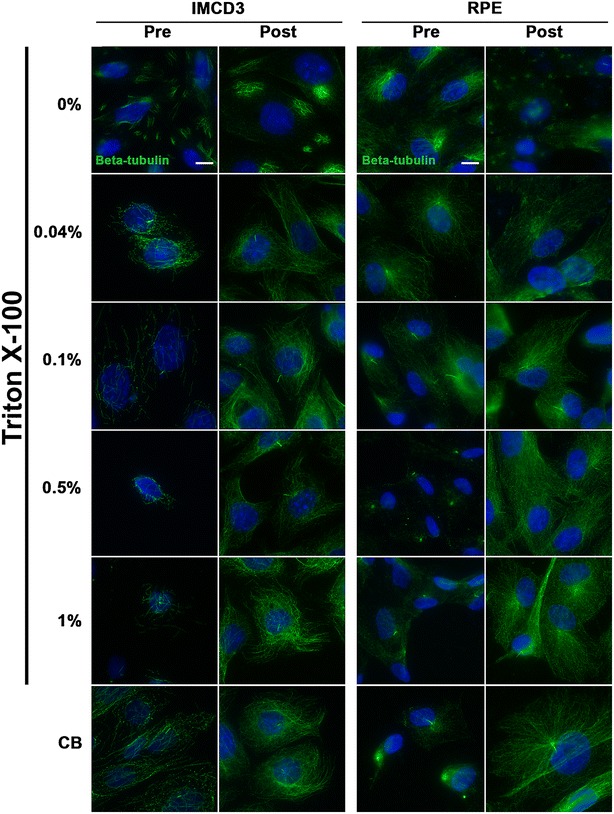
Permeabilization before or after PFA fixation affects microtubule immunolabeling in IMCD3 and RPE cells. Cells were either (1) washed with a permeabilizing solution before being fixed with PFA-S, or (2) fixed with PFA-S, and then permeabilized with solutions of different strength. Cilia immunolabeling was assessed with a β-tubulin antibody (*green*) and Hoechst dye for nuclear staining (*blue*). No permeabilization resulted in little or absent cilia labeling and sporadic microtubule cytoskeleton staining. Permeabilization of any strength resulted in robust cilia labeling and microtubule staining. Permeabilization strength was negatively correlated with the preservation of microtubule labeling, although CB which contains 0.5% Triton X-100 appears to offer some benefit to microtubule preservation. Permeabilization done after cells were already fixed did not affect microtubule or cilia immunolabeling. *Scale bars* 10 µm

Microtubules as assessed with β-tubulin antibody appeared to be preserved, though to different extents, with all fixation methods tested (PFA-S; PFA-PBS; MeOH; CB-PFA; CB → MeOH; CB → CB-PFA; CB → CB-PFA → MeOH) in RPE and IMCD3 cells (Fig. 1; Table 1). Moreover, β-tubulin antibody labeled the cilium to varying degrees in the tested fixation methods (Fig. 1; Table 1). Methods that did not include a pre-CB wash (PFA-S, PFA-PBS, MeOH, and CB-PFA) yielded positive staining throughout the cell cytoplasm and at the cilium. But, especially when fixed with PFA-S or PFA-PBS, the bright labeling of microtubules in the cytoplasm often obscured the presence of a cilium. The opposite was found with fixation methods that included a CB wash before cells were fixed (CB → MeOH, CB → CBPFA, or CB → CBPFA → MeOH). Washing cells with CB (Fig. 1) or with detergent (Fig. 2) yielded little cytoskeletal microtubule staining, and thus, made cilia immunolabeling more prominent. Caution is needed, however, when using a high concentration of Triton X-100 when applying a pre-wash because it often led to loss of cells on the coverslip. Not using Triton X-100 at all was also not an option, as evidence by our 0% Triton X-100 treatment groups, which showed only sporadic microtubule staining and no cilia localization with the β-tubulin antibody (Fig. 2).

**Table 1:** Summary of fixation methods and their effects on cilia marker immunolabeling

Antibodies	Cilia staining						
	PFA-S	PFA/PBS	MeOH	CBPFA	CB MeOH	CB CBPFA	CB CBPFA MeOH
*IMCD3*							
β-tubulin	+	+	+	+	++	+++	+++
Acetylated-α- tubulin	+++	+++	++	+++	+++	+++	+++
Detyrosinated tubulin	+++	+++	+++	+	+	+++	+++
Polyglutamylated tubulin	+	+	+/−	+++	+++	+++	+++
AC3	+	+	−	−	−	−	−
Arl13b	+++	+++	+	+++	−	−	−
CSPP1	+/−	+/−	–	++	–	+	+++
IFT20	−	−	−	−	+	+	+
*RPE*							
β-tubulin	+/−	+	+	+	++	+++	+++
Acetylated-α-tubulin	+++	+++	+++	+++	+++	+++	+++
Detyrosinated tubulin	+++	+++	+++	+++	+++	+++	+++
Polyglutamylated tubulin	++	+++	+/−	++	+++	+/−	+++
AC3	−	−	−	−	−	−	−
Arl13b	+++	+++	−	+++	−	−	−
CSPP1	+	+	−	+++	−	+++	+
IFT20	−	−	−	+/−	+	++	+++

−, no cilia staining; +/−, some, but not all cells have cilia staining; +, cilia staining is present, but not obvious (i.e. high background); ++, easily noticeable cilia staining; +++, cilia staining is bright with low background

Actin stress fibers as assessed with phalloidin staining were not preserved when using any fixation method that included the use of methanol (MeOH, CB → MeOH, or CB → CB-PFA → MeOH), but were intact with PFA-S, PFA-PBS, CB-PFA, or CB → CB-PFA fixation methods (Fig. 1).

### Different fixation methods affect cilia marker immunolabeling localizations

To test these various fixation methods and their effects on ciliary compartment labeling, we chose antibodies that labeled the structural components of the microtubule axoneme (acetylated α-tubulin, detyrosinated tubulin, and polyglutamylated tubulin), proteins bound to the axoneme (CSPP1 and IFT20), and ciliary membrane proteins (AC3 and Arl13b).

**Fig. 3:**
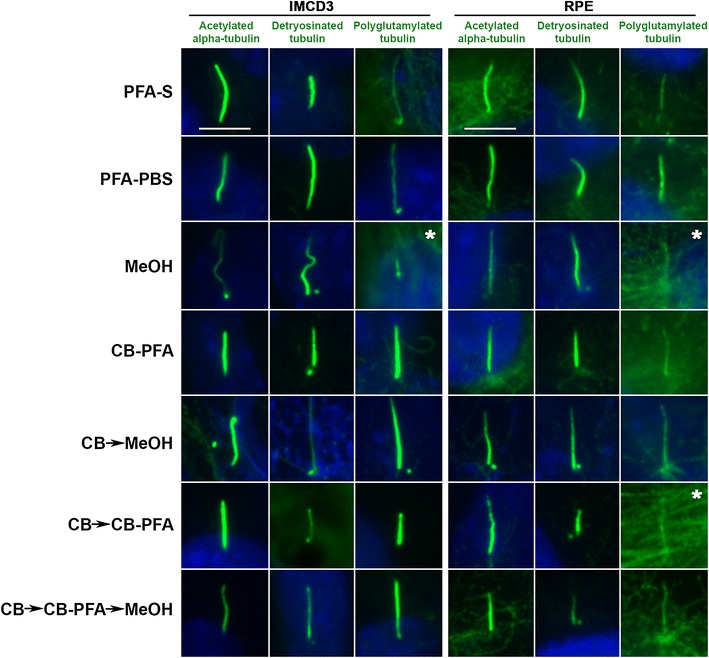
Different fixation methods and its effects on structural tubulin post-translational protein immunolabeling of cilia in IMCD3 and RPE cells. IMCD3 and RPE cells were fixed with various fixation methods, and then probed with: acetylated-α-tubulin, detyrosinated tubulin, or polyglutamylated tubulin antibody. Cilia staining was preserved in all groups to varying levels of success. *Panels denoted by asterisk* have rare and difficult to identify cilia immunolabeling. *Scale bars* 5 µm

All three antibodies tested that were targeted to post-translational modifications on tubulin (acetylated α-tubulin, detyrosinated tubulin, and polyglutamylated tubulin) produced cilia labeling to varying degrees of success when subjected to different fixation methods (Fig. 3; Table 1). We observed that fixation methods that employed the use of methanol (MeOH, CB → MeOH, and CB → CBPFA → MeOH) tended to increase the presence of centrosomal staining at the base of cilia in both IMCD3 and RPE cells (Fig. 3). While cilia labeling appears prominently for the three antibodies in most cases (Fig. 3), staining showed a difference especially with polyglutamylated tubulin antibody (Fig. 3). In IMCD3 and RPE cells, both acetylated α-tubulin and detyrosinated tubulin showed prominent cilia labeling across all fixation methods tested (Fig. 3). When probing for polyglutamylated tubulin, however, the microtubule labeling with this antibody was intense, making it difficult to find and identify cilia, especially in methanol-treated cells (for both IMCD3 and RPE cells) and in the CB → CB-PFA group (in RPE cells) (Fig. 3).

**Fig. 4:**
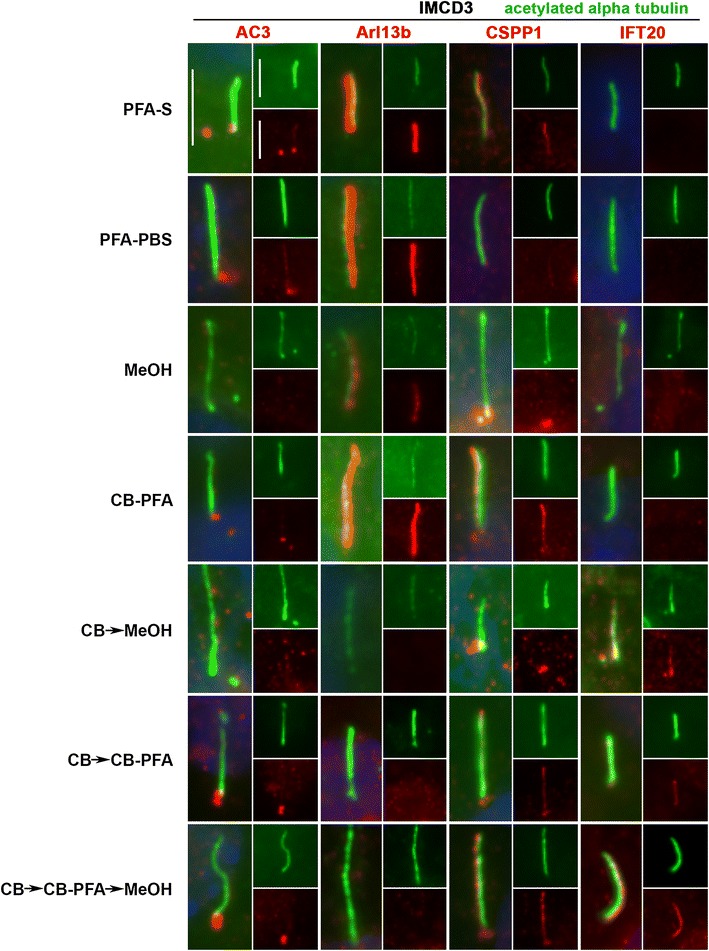
Different fixation methods affect cilia marker immunolabeling in IMCD3 cells. IMCD3 cells were fixed with various fixation methods and then probed with AC3, Arl13b, CSPP1, and IFT20 antibodies (*red*). All coverslips were also co-stained with acetylated-α-tubulin (*green*) for cilia labeling and Hoechst for nuclear labeling (*blue*). *Insets* are separated channel images at 50% of the original image. Generally, cilia immunolabeling was best observed for the membrane-localizing proteins (AC3 and Arl13b) in the PFA-S group, while axoneme-localizing proteins (CSPP1 and IFT20) were best preserved in cilia treated with CB fixation methods. *Scale bars* 5 µm

**Fig. 5:**
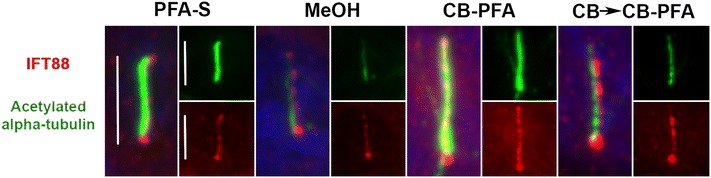
Different fixation methods affect IFT88 immunolabeling at cilia in IMCD3 cells. IMCD3 cells were subjected to various fixation methods and then co-labeled with IFT88 (*red*) and acetylated-α-tubulin (*green*). All coverslips were also stained with Hoechst for nuclear labeling (*blue*). IFT88 labels the cilium in all fixation methods tested. *Scale bars* 5 µm

**Fig. 6:**
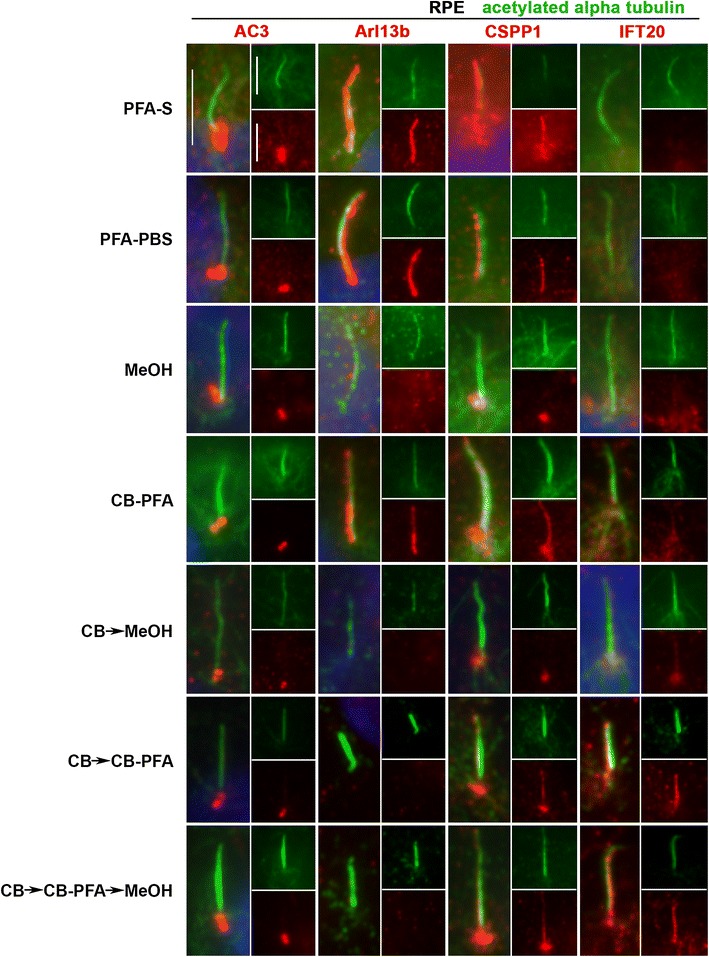
Different fixation methods affect cilia marker immunolabeling in RPE cells. RPE cells were subjected to various fixation methods and then probed with AC3, Arl13b, CSPP1, and IFT20 antibodies (*red*). All coverslips were also co-stained with acetylated-α-tubulin (*green*) for cilia labeling and Hoechst for nuclear labeling (*blue*). *Insets* are separated channel images at 50% of the original image. Generally, cilia immunolabeling was best observed for the membrane-localizing proteins (AC3 and Arl13b) in the PFA-S group, while axoneme-localizing proteins (CSPP1 and IFT20) were best preserved in cilia treated with CB fixation methods. *Scale bars* 5 µm

In IMCD3 cells, PFA-S fixation reliably revealed ciliary labeling with the Arl13b antibody, and to a lesser extent, preserved less distinct cilia immunolabeling with the CSPP1 antibody in some cells (Fig. 4; Table 1). PFA-S fixation also preserved prominent AC3 labeling at the mother and daughter centrioles, and could also be observed less intensely in some cilia (Fig. 4). PFA-S fixation did not yield ciliary or basal body immunolabeling for IFT20 (Fig. 4). IFT88, unlike other axoneme proteins tested, labeled cilia in all fixation methods tested (Fig. 5). These PFA-S observations were identical in RPE cells, including the labeling for CSPP1 in cilia not being seen in all cells and not as distinct (Fig. 6; Table 1). Results obtained with PFA-PBS fixation were indiscernible from PFA-S fixation for both cell types (Figs. 4, 6).

While many laboratories have had success with MeOH fixation, our results demonstrated that MeOH fixation was the least reliable and replicable fixation method attempted. With MeOH fixation, cilia labeling could be observed for Arl13b in IMCD3 cells (Fig. 4), but not in RPE cells (Fig. 6). AC3 was localized at mother and daughter centrioles with MeOH fixation in IMCD3 (Fig. 4) and RPE cells (Fig. 6). CSPP1 only immunolabeled centrioles in IMCD3 (Fig. 4) and RPE (Fig. 6) cells with MeOH fixation. For IFT20 immunolabeling with MeOH fixation, no cilia or centrosomal labeling was observed for either cell type (Figs. 4, 6).

Of all the variations using CB fixation, all antibodies produced similar immunolabeling patterns in both cell lines. AC3 only labeled centrioles (Figs. 4, 6; Table 1). Arl13b did not label cilia in the CB → MeOH, CB → CB-PFA or CB → CB-PFA → MeOH fixation groups in either cell line, but CB-PFA fixation did preserve cilia immunolabeling in both cell lines similar to PFA (Figs. 4, 6; Table 1). CSPP1 produced distinct centrosome labeling with all the CB variation methods and yielded cilia immunolabeling in IMCD3 and RPE cells with varying levels of intensity (Figs. 4, 6; with CB-PFA being the most consistent across cell lines). The exception was CB → MeOH fixation which did not label cilia in either cell type (Figs. 4, 6; Table 1). IFT20 showed unreliable cilia labeling with CB-PFA fixation, but produced distinct cilia staining in all other CB fixation methods (CB → MeOH, CB → CBPFA, and CB → CBPFA → MeOH) tested (Figs. 4, 6; Table 1).

### Different fixation methods affect mitotic figure labeling

**Fig. 7:**
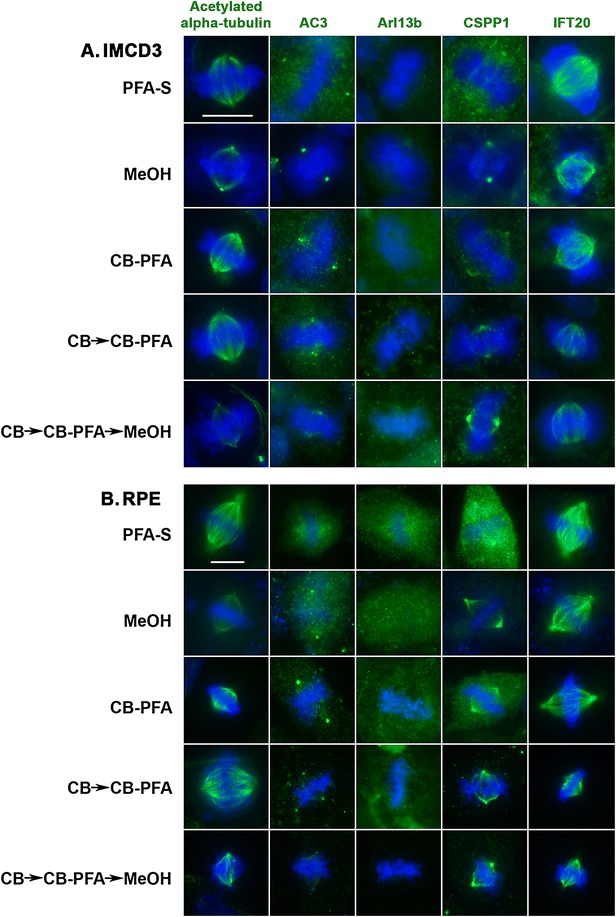
Different fixation methods affect mitotic figure labeling in IMCD3 and RPE cells. IMCD3 and RPE cells were fixed with various fixation methods, and then probed with acetylated-α-tubulin to establish images of metaphase mitotic figures when subjected to different fixation methods. Separate coverslips of cells were subjected to labeling with either a membrane-bound cilia marker (AC3, Arl13b; *green*) or an axoneme-bound cilia marker (CSPP1, IFT20; *green*). All cells were stained with Hoechst for nuclear labeling (*blue*). **A** In IMCD3 cells, Arl13b did not immunolabel mitotic figures, but CSPP1, IFT20, and AC3 labeled mitotic spindles and/or centrosomes depending on the fixation method used. MeOH-treated IMCD3 cells produced the most intense centrosomal immunolabeling for CSPP1. **B** In RPE cells, Arl13b also did not label mitotic figures with any tested fixation method. *Scale bars* 10 µm

Microtubules that make up the mitotic spindles, as assessed by acetylated α-tubulin immunostaining, were preserved in both cell lines with all fixation methods, but with noticeable differences (Fig. 7). IMCD3 cells that were treated with methanol (MeOH and CB → CBPFA → MeOH) have distorted DNA labeling (Hoechst) and less intense mitotic spindle staining. However, MeOH fixation, for both IMCD3 and RPE cells, showed the most distinct and reproducible centrosomal immunolabeling with acetylated α-tubulin antibody (Fig. 7). All other fixation groups tested showed some centrosomal labeling, but this was only found in a minority of cells and displayed much less intense labeling at centrosomes (Fig. 7).

With the exception of IFT20, mitotic figures were largely obscured in a poorly defined haze when cells were fixed with PFA-S (Fig. 7). IMCD3 and RPE cells fixed with MeOH revealed intense centrosomal labeling with CSPP1, mitotic spindle labeling with IFT20, but no distinct immunolabeling with Arl13b (Fig. 7). Methanol-fixed RPE cells also showed centrosomal staining in mitotic figures with AC3 antibody. CB fixation methods produced consistent immunolabeling across both cell lines for the antibodies tested. AC3 labeled positive for centrosomes and weakly for mitotic spindles in RPE and IMCD3 cells, while CB fixation techniques did not reveal any mitotic figure labeling with Arl13b for either cell line (Fig. 7). CSPP1 immunolabeled centrosomes, and both CSPP1 and IFT20 labeled mitotic spindles in both cell lines when fixed with either variation of CB fixation. For this set of experiments, we excluded the PFA–PBS group since we do not observe any differences between PFA-S and PFA–PBS. We also excluded the CB → MeOH group because it was less reliable and resulted in a higher percentage of cell loss from the coverslips.

### Different fixation methods affect IFT20 labeling at *cis*-Golgi

**Fig. 8:**
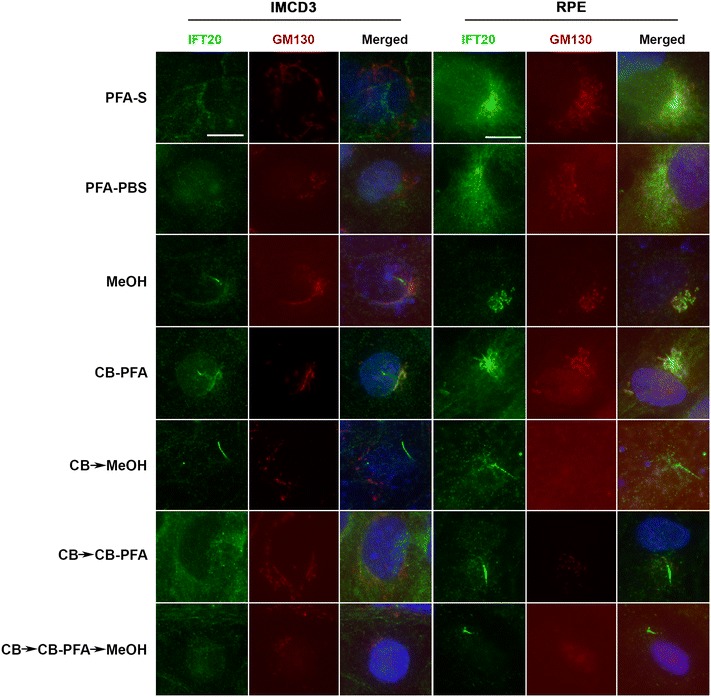
Different fixation methods affect IFT20 labeling of *cis*-Golgi in IMCD3 and RPE cells. IMCD3 and RPE cells were subjected to various fixation methods and then co-stained with IFT20 (*green*) and GM130 (a *cis*-Golgi marker; *red*). All coverslips were also stained with Hoechst for nuclear labeling (*blue*). Co-labeling was only observed in the methanol and CB-PFA groups. *Scale bars* 10 µm

IFT20 co-labels with GM130, a *cis*-Golgi marker, in IMCD3 and RPE cells when cells are fixed with methanol or CB-PFA (Fig. 8). Other fixation groups tested yielded either non-distinct or absent IFT20 staining (Fig. 8) around the cell nucleus (PFA-S, PFA-PBS, CB → MeOH, CB → CB-PFA, and CB → CB-PFA → MeOH). GM130 immunolabeling was also affected by the fixation method used, and was often observed to have a washed out appearance in the CB treatment groups, especially in RPE cells (Fig. 8).

## Discussion

It is becoming increasingly clear that cilia proteins can also have extraciliary localizations and functions [18, 26, 37, 38]. Further work is needed to determine which ciliary proteins have extraciliary functions, and whether these extraciliary functions of cilia proteins contribute to the pathology of ciliopathies. If so, what symptoms can be attributed to defective cilia, and what symptoms are related to defects at extraciliary sites? As these questions are explored and the function of these extraciliary sites for ciliary proteins is revealed, an understanding of how fixation affects immunofluorescent labeling is warranted. As described earlier, an inadequate exploration of fixation techniques has led to contradictory conclusions by different laboratories. CSPP1 has been reported to be exclusively a centrosomal protein [33], exclusively an axonemal protein [34], or both a centrosomal and an axonemal protein [20] depending upon the fixation method used. Even widely used antibodies like anti-acetylated α-tubulin can produce differing results depending on the method of fixation utilized. The acetylated α-tubulin antibody immunolabels both cilia and centrosomes in MeOH-fixed cells, but in PFA-fixed cells, immunolabeling reveals only cilia with a few cells exhibiting centrosomal labeling. Therefore, here we wanted to conduct a comprehensive and systematic examination of the effects of fixation on ciliary marker immunolabeling.

The goal of this study was to establish a reliable and flexible fixation technique that could concurrently visualize cilia, microtubules, and actin stress fibers (as assessed with phalloidin). However, current results demonstrate that each fixation method has advantages and disadvantages, and it may be necessary to use different fixation methods when studying different areas of a cell even when studying the same protein. For example, PFA-S fixation reveals AC3 to be at the primary cilium in IMCD3 cells, but when used to look at mitotic figures, AC3 only labels centrioles. However, if the goal was to examine AC3 immunolabeling at mitotic figures and not cilia, then any of the CB fixation methods would be preferable over PFA-S as these methods reveal AC3 localization to the mitotic spindles as well as the centrosomes; a finding that is novel to our knowledge. Unfortunately, the CB fixation methods result in a loss of primary cilia staining with the AC3 antibody, so we were unable to find one fixation protocol that could reveal all of the ciliary and extraciliary localizations for AC3. Therefore, the fixation method used will be dependent on the antibody and the cellular structure that is being studied.

By utilizing the two workhorse cells in the cilia field, mouse IMCD3 and human RPE cells, we have found that for some cilia markers, immunolabeling patterns are dependent on the fixation technique used and can be cell type specific. Comparison of two different cell types allows for the examination of potential cell type differences, but attention is warranted as to how much we can extrapolate from these findings. IMCD3 and RPE cells are both epithelial type cells, but IMCD3s are mouse kidney cells whereas RPE cells are derived from human eye. Therefore, any difference we see between RPE and IMCD3 protein expression patterns could be due to cell type differences, species differences, or antibody binding differences. It is beyond the scope of this manuscript to examine more cell types to tease out these differences, especially since we wanted to use already established ciliary cell lines. However, our results indicate that each cell line utilized should undergo some degree of troubleshooting for the best fixation methodology to use with the hope that our approach provides some directed guidance.

Of the four ciliary markers we examined, Arl13b and AC3 were directed against mouse epitopes, and IFT20 and CSPP1 were directed against human epitopes. Arl13b was the only monoclonal antibody with the three remaining antibodies being polyclonal antibodies. Differences in human vs. mouse reactive epitopes among antibodies can sometimes yield staining differences, but we did not observe any in our data when comparing IMCD3 and RPE cells. For example, CSPP1 is directed against human CSPP1 protein, but it does not appear to label human RPE cells any better than in the mouse IMCD3 cell line. As a whole, our data comparing cilia protein immunostaining in IMCD3 and RPE cells look remarkably similar.

When we applied a 10-min fixation incubation time for all fixation methods to both cell types, we found overall that RPE cells maintained a more intact microtubule cytoskeleton. This may not only be due to cell type-specific differences in structural resilience of the microtubule cytoskeleton to fixation agents, but may also be due to the fact that IMCD3 cells are smaller than RPE cells. Thus, it may be necessary to empirically determine the proper fixation method and incubation period for each individual cell type and fixation method used in a study. Here, by applying the same 10-min incubation period for all experiments, we hoped to gain a general understanding of how various fixation agents affected the immunolabeling pattern for some popularly used cilia markers.

We fixed our cells at three different temperatures: −20 °C for methanol fixation, room temperature for PFA fixation techniques, and 37 °C for cytoskeletal buffer-based techniques. We chose these temperatures based on what is most appropriate for each individual fixation agent. Methanol must be used cold. PFA is traditionally used at room temperature. CB and CB-PFA were used at 37 °C because Weisenberg showed that in vitro tubulin polymerizes better at more physiological temperatures [31]. However, the temperature at which a cell is fixed may influence the resulting immunolabeling pattern, since colder fixation temperatures may benefit proteins that are susceptible to rapid degradation. While it is possible that the different temperatures of wash buffers and fixatives utilized in our experiments may affect cilia immunostaining, we avoided straying from conventional temperatures used for these fixation agents to stay consistent with what is already done in the field. However, temperature of washes and fixation could be an important variable for consideration in examining ciliary and extraciliary immunolabeling localizations.

We examined a variety of different fixation techniques to see which methods were amenable to actin stress fiber staining by phalloidin, microtubule and cilia staining by β-tubulin, and three antibodies directed towards tubulin post-translational modifications. We found that acetylated α-tubulin labeled microtubules comparably with all fixation methods used, but the MeOH-fixed cells had the distinction of having much more prominent centriolar labeling at the base of cilia. Using a CB wash before fixing cells also offers a more “washed out” cytoplasmic appearance, offering greater contrast between microtubules and a cleared cytoplasm. Phalloidin labeling was preserved in fixation methods that did not use MeOH (PFA-S, CB → CB-PFA, and CB-PFA), but was damaged in any method that used MeOH regardless of whether MeOH was used alone or after being fixed with CB and PFA. Thus, CB does not damage phalloidin staining of actin stress fibers, since they were preserved in both the CB-PFA and CB → CB-PFA fixation groups. But PFA-S did not protect the phalloidin epitope from MeOH, since the CB → CB-PFA → MeOH fixation group lost actin stress fiber labeling as assessed by phalloidin. Finally, we saw no benefit in using MeOH over PFA since PFA fixation techniques offered more replicable results and have the advantage that it does not damage the phalloidin epitope.

In this manuscript, we purposely chose commonly used and published ciliary markers that are already established by others in the field. We also are cautious to not make any functional claims about any protein. We do note the location of each protein as it differs from one fixation method to the other to illustrate that fixation methods can affect the staining pattern of an antibody. But we acknowledge that each finding in this paper must be empirically verified to determine the functional significance of these localizations through knockout or knockdown studies to draw any conclusions about the function of a protein and the specificity of an antibody.

**Fig. 9:**
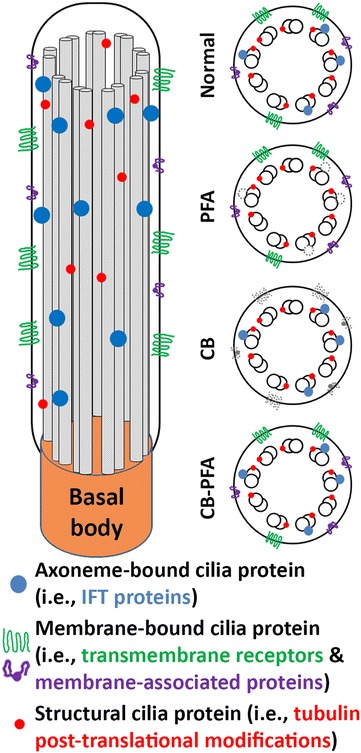
Summary of general fixation guidelines. PFA fixation preserved most membrane-localized cilia proteins and tubulin post-translational modification immunolabeling. Application of a cytoskeletal buffer (CB) wash or pre-fixation permeabilization with Triton X-100 (not shown) washed away many membrane-cilia proteins, but revealed axonemal-cilia and post-translational modification immunolabeling. Fixation in PFA prepared in CB (CB-PFA) almost always preserved membrane-cilia proteins, axonemal-cilia proteins, and post-translational modification of tubulin immunolabeling

When our assortment of fixation methods was tested against our selection of cilia markers, a pattern was quickly noticed that may be generally applicable to other ciliary antibodies. The cilia markers appear to be able to be separated into three distinct groups: (1) tubulin structural markers, (2) membrane-associated proteins, and (3) axoneme-associated proteins (Fig. 9). All three post-translational modification antibodies and the β-tubulin antibody immunolabeled cilia regardless of the fixation method used although some were more effective than others. It appears that structural proteins or at least epitopes for these structural proteins are stable against PFA, methanol, and variations of cytoskeletal buffer fixation techniques. Conversely, membrane-bound proteins (AC3 and Arl13b) were best preserved at cilia when immunolabeled after PFA fixation. Arl13b cilia immunolabeling was also observed in IMCD3 and RPE cells that were fixed with MeOH or CB-PFA. We surmise that by combining CB with PFA, to make a 4% CB-PFA fixation solution, we were able to combine the cytoskeletal stabilizing benefits of CB along with the quick fixation properties of PFA, preserving Arl13b localization at cilia before any harsh membrane-washing could take place. But fixation methods that used a CB pre-wash appear to have washed away the Arl13b and AC3 cilia labeling. It is interesting that while CB-PFA fixation was able to preserve Arl13b labeling at cilia, it could not preserve AC3 cilia immunolabeling, suggesting that this method has its limitations. Last, axoneme-associated proteins (CSPP1 and IFT20) unreliably immunolabel cilia when fixed with PFA, but are revealed as cilia markers when cells are fixed with either of the CB fixation methods. This suggests that either (1) CB stabilizes the epitope of axoneme-associated proteins, or (2) CB washes away the cilia membrane to allow antibodies to access the axoneme-associated proteins for labeling. The former hypothesis is more likely since tubulin structural markers, which reside along the axoneme, are accessible without the use of CB. In addition, when we applied our fixation guidelines to IFT88, we found that IFT88 defies our categorization as it immunolabels cilia when fixed with PFA alone, methanol, or cytoskeletal buffer-based techniques. However, we observed that IFT88 labels the cilium more distinctly when CB is used, so while CB may not be necessary to observe IFT88-positive cilia, CB can be used to help improve cilia immunolabeling.

The MeOH-fixed groups were the least replicable of the fixation methods we tested, and therefore, were the most difficult groups to characterize. Moreover, MeOH is still not an ideal fixation agent for our goals as it results in distorted DNA labeling, and obscures the epitope for phalloidin. Finally, MeOH fixation does not fit our categorization of membrane-localizing and axoneme-localizing proteins as it preserves some but not all proteins from each group.

Our goals for this paper were to demonstrate the importance of fixation on cilia protein immunolabeling, and to establish guidelines that may help others optimize their fixation protocols for studying cilia proteins at ciliary and extraciliary sites. While we could not analyze all the many extraciliary sites now known in the field to be important for cilia proteins, we did choose two sites to examine our fixation methods in (1) mitotic figures and (2) *cis*-Golgi. Mitotic figures are similar to cilia in that they consist of microtubules that extend from centrosomes, so it is not surprising that many proteins that localize to the cilium and mother/daughter centrioles are also found at mitotic figures. Microtubules of mitotic figures proved to be similar to microtubules of the cytoskeleton in that a tubulin structural marker, acetylated α-tubulin, was able to immunolabel them with all fixation methods tested. Cilia protein labeling at mitotic figures was observed as expected with varying degrees of distinctiveness depending on the fixation method used: CSPP1 and IFT20 labeled the microtubules, AC3 labeled the centrosomes, and Arl13b did not label any part of the mitotic figure. Interestingly, AC3 appears to label the microtubules of the mitotic figure in CB fixation groups, a finding that is novel to our understanding, and requires further investigation to verify. The methanol and CB groups revealed less background staining when compared to the PFA-S group, making the microtubule and centrosome staining more distinct.

Fixation methods also affected IFT20 staining at the Golgi. IFT20 is known to localize at the Golgi and has been observed at the Golgi in PHEM-treated cells (another version of a cytoskeletal buffer) and methanol-treated cells [24]. Our results were similar. IFT20 co-localized with GM130, a *cis*-Golgi marker, only when IMCD3 and RPE cells were treated with methanol or CB-PFA. Fixation methods also appear to affect GM130 staining as GM130 labeling was often lost in the CB fixation groups. Altogether, these results show that not unlike at the cilium, cilia protein localization at extraciliary sites is also affected by the fixation method used.

When looking at the totality of our data, no one fixation method can be used to preserve all groups of cilia proteins, but CB-PFA comes close. CB-PFA is suitable for use when staining for microtubules, phalloidin-stained actin stress fibers, most cilia proteins we tested, and for at least two extraciliary sites (mitotic figures and IFT20 labeled Golgi). CB-PFA preserved Arl13b (a membrane-associated protein) and CSPP1 (an axoneme-associated protein) staining at cilia, but it appears to wash away AC3 cilia labeling, and it is not a reliable method for IFT20 cilia staining. This discrepancy in cilia immunolabeling within each cilia protein group is not straightforward, but intriguing. Could AC3 be more vulnerable to being washed off the cilia membrane than Arl13b? Are there different compartments of varying stabilities on a ciliary membrane much like lipid rafts exist as distinct compartments on the plasma membrane? Finally, it is also noteworthy that while IFT20 lightly and unreliably labels cilia in CB-PFA-fixed cells, cilia staining with ITF20 is dramatically improved when a CB pre-wash is utilized. A CB pre-wash is harsher than fixing with CB-PFA alone, so this might suggest that the location of IFT20 on the ciliary axoneme requires harsher permeabilization to access than CSPP1? Such questions require further study and may be elucidated through an understanding of different fixation techniques.

## Conclusions

As the cilia field moves forward in examining non-ciliary sites and as the field continues to expand, it is useful to consider and appreciate alternate fixation methods. Our findings have revealed a level of complexity in cilia proteins that suggests cilia proteins can be separated into different groups and each group benefits from different fixation methods. Structural proteins like β-tubulin and the various post-translational modification markers are resilient to various fixation methods. Cilia-membrane proteins are best fixed with PFA alone, but axonemal proteins are often obscured with PFA fixation and benefit from CB fixation (Fig. 9). The most universal method we found was the CB-PFA method (however, there can be exceptions; Fig. 9). CB-PFA appears to be the most versatile of the fixation methods we tested as it preserves the microtubule cytoskeleton, phalloidin labeled actin stress fibers, and most cilia labeling. This method can also easily be adjusted to reveal more distinct cilia labeling by adding a CB wash to the protocol, though this can come at a cost (e.g., loss of Arl13b labeling). Although there are caveats for using any fixation method, we advocate the usage of cytoskeleton buffer as a starting point, since the primary cilium is essentially a cytoskeletal organelle composed of microtubules extending from the cytoskeleton of the cell.

## Abbreviations

AC3: adenylyl cyclase 3
Arl13b: ADP-ribosylation factor-like protein 13b
CB: cytoskeletal buffer
CB-PFA: 4% PFA prepared in cytoskeletal buffer
CSPP1: centrosome and spindle pole associated protein 1
GM130: Golgi matrix protein 130
IFT: intraflagellar transport
IFT20: intraflagellar transport protein 20
IFT88: intraflagellar transport protein 88
IMCD3: mouse inner medullary collecting duct 3 cells
MeOH: methanol
PBS: phosphate-buffered saline
PFA: paraformaldehyde
PFA-S: paraformaldehyde–sucrose
Post-PFA-S: treatment group where cells were fixed with PFA-S before being permeabilized
Pre-PFA-S: treatment group where cells were permeabilized before being fixed with PFA-S
RPE: human retinal pigmented epithelial cells

## References

[1] Hildebrandt F, Benzing T, Katsanis N (2011). Ciliopathies. N Engl J Med.

[2] Satir P, Christensen ST (2007). Overview of structure and function of mammalian cilia. Annu Rev Physiol.

[3] Satir P, Christensen ST (2008). Structure and function of mammalian cilia. Histochem Cell Biol.

[4] Hsiao YC, Tuz K, Ferland RJ (2012). Trafficking in and to the primary cilium. Cilia..

[5] Kim S, Dynlacht BD (2013). Assembling a primary cilium. Curr Opin Cell Biol.

[6] Kobayashi D, Takeda H (2012). Ciliary motility: the components and cytoplasmic preassembly mechanisms of the axonemal dyneins. Differentiation..

[7] Jin X, Mohieldin AM, Muntean BS, Green JA, Shah JV, Mykytyn K, Nauli SM (2014). Cilioplasm is a cellular compartment for calcium signaling in response to mechanical and chemical stimuli. Cell Mol Life Sci.

[8] Lechtreck KF (2015). IFT-cargo interactions and protein transport in cilia. Trends Biochem Sci.

[9] Waters AM, Beales PL (2011). Ciliopathies: an expanding disease spectrum. Pediatr Nephrol.

[10] Wheatley DN (2005). Landmarks in the first hundred years of primary (9+0) cilium research. Cell Biol Int.

[11] Zimmerman K (1898). Beitrage zur kenntniss einiger drusen und epithelien. Arch Mikrosk Anat..

[12] Barnes BG (1961). Ciliated secretory cells in the pars distalis of the mouse hypophysis. J Ultrastruct Res.

[13] Poole CA, Flint MH, Beaumont BW (1985). Analysis of the morphology and function of primary cilia in connective tissues: a cellular cybernetic probe?. Cell Motil..

[14] Singla V, Reiter JF (2006). The primary cilium as the cell’s antenna: signaling at a sensory organelle. Science.

[15] Marshall WF, Nonaka S (2006). Cilia: tuning into the cell’s antenna. Curr Biol.

[16] Green JA, Mykytyn K (2014). Neuronal primary cilia: an underappreciated signaling and sensory organelle in the brain. Neuropsychopharmacology..

[17] Berbari NF, O’Connor AK, Haycraft CJ, Yoder BK (2009). The primary cilium as a complex signaling center. Curr Biol.

[18] Vertii A, Bright A, Delaval B, Hehnly H, Doxsey S (2015). New frontiers: discovering cilia-independent functions of cilia proteins. EMBO Rep.

[19] Patzke S, Hauge H, Sioud M, Finne EF, Sivertsen EA, Delabie J, Stokke T, Aasheim HC (2005). Identification of a novel centrosome/microtubule-associated coiled-coil protein involved in cell-cycle progression and spindle organization. Oncogene.

[20] Patzke S, Redick S, Warsame A, Murga-Zamalloa CA, Khanna H, Doxsey S, Stokke T (2010). CSPP is a ciliary protein interacting with Nephrocystin 8 and required for cilia formation. Mol Biol Cell.

[21] Sternemalm J, Geimer S, Frikstad KA, Schink KO, Stokke T, Patzke S (2015). CSPP-L associates with the desmosome of polarized epithelial cells and is required for normal spheroid formation. PLoS ONE.

[22] Zhu L, Wang Z, Wang W, Wang C, Hua S, Su Z, Brako L, Garcia-Barrio M, Ye M, Wei X, Zou H, Ding X, Liu L, Liu X, Yao X (2015). Mitotic protein CSPP1 interacts with CENP-H protein to coordinate accurate chromosome oscillation in mitosis. J Biol Chem.

[23] Barral DC, Garg S, Casalou C, Watts GF, Sandoval JL, Ramalho JS, Hsu VW, Brenner MB (2012). Arl13b regulates endocytic recycling traffic. Proc Natl Acad Sci USA.

[24] Follit JA, Tuft RA, Fogarty KE, Pazour GJ (2006). The intraflagellar transport protein IFT20 is associated with the Golgi complex and is required for cilia assembly. Mol Biol Cell.

[25] Patzke S, Stokke T, Aasheim HC (2006). CSPP and CSPP-L associate with centrosomes and microtubules and differently affect microtubule organization. J Cell Physiol.

[26] Hsiao YC, Tong ZJ, Westfall JE, Ault JG, Page-McCaw PS, Ferland RJ (2009). Ahi1, whose human ortholog is mutated in Joubert syndrome, is required for Rab8a localization, ciliogenesis and vesicle trafficking. Hum Mol Genet.

[27] Rao Y, Hao R, Wang B, Yao TP (2014). A Mec17-Myosin II effector axis coordinates microtubule acetylation and actin dynamics to control primary cilium biogenesis. PLoS ONE.

[28] Hong H, Kim J, Kim J (2015). Myosin heavy chain 10 (MYH10) is required for centriole migration during the biogenesis of primary cilia. Biochem Biophys Res Commun..

[29] Delgehyr N, Meunier A, Faucourt M, Bosch Grau M, Strehl L, Janke C, Spassky N (2015). Ependymal cell differentiation, from monociliated to multiciliated cells. Methods Cell Biol.

[30] Osborn M, Weber K (1977). The display of microtubules in transformed cells. Cell.

[31] Weisenberg RC (1972). Microtubule formation in vitro in solutions containing low calcium concentrations. Science.

[32] Bershadsky AD, Gelfand VI, Svitkina TM, Tint IS (1978). Microtubules in mouse embryo fibroblasts extracted with Triton X-100. Cell Biol Int Rep.

[33] Akizu N, Silhavy JL, Rosti RO, Scott E, Fenstermaker AG, Schroth J, Zaki MS, Sanchez H, Gupta N, Kabra M (2014). Mutations in CSPP1 lead to classical Joubert syndrome. Am J Hum Genet.

[34] Tuz K, Bachmann-Gagescu R, O’Day DR, Hua K, Isabella CR, Phelps IG, Stolarski AE, O’Roak BJ, Dempsey JC, Lourenco C (2014). Mutations in CSPP1 cause primary cilia abnormalities and Joubert syndrome with or without Jeune asphyxiating thoracic dystrophy. Am J Hum Genet.

[35] Withers GS, Banker G, Banker G, Goslin K (2002). Characterizing and studying neuronal cultures. Culturing nerve cells.

[36] Schneider CA, Rasband WS, Eliceiri KW (2012). NIH Image to ImageJ: 25 years of image analysis. Nat Methods.

[37] Boehlke C, Janusch H, Hamann C, Powelske C, Mergen M, Herbst H, Kotsis F, Nitschke R, Kuehn EW (2015). A cilia independent role of Ift88/Polaris during cell migration. PLoS ONE.

[38] Boehlke C, Kotsis F, Buchholz B, Powelske C, Eckardt KU, Walz G, Nitschke R, Kuehn EW (2013). Kif3a guides microtubular dynamics, migration and lumen formation of MDCK cells. PLoS ONE.

